# Study on the Corrosion Behavior of Cemented Organic Soil in Dianchi Lake, China

**DOI:** 10.3390/ma16175951

**Published:** 2023-08-30

**Authors:** Wenlian Liu, Jing Cao, Yunfei Song, Sugang Sui, Hanhua Xu, Yongfa Guo, Wenyun Ding, Siyang Huang

**Affiliations:** 1 Yunnan Key Laboratory of Geotechnical Engineering and Geohazards, Kunming 650051, China; liuwenlian@zskk1953.com (W.L.); suisugang2@zskk1953.com (S.S.); xuhanhua@zskk1953.com (H.X.); 2 Faculty of Civil Engineering and Mechanics, Kunming University of Science and Technology, Kunming 650500, China; 20202210067@stu.kust.edu.cn; 3 Kunming Survey, Design and Research Institute Co., Ltd. of CREEC, Kunming 650200, China; guoyf@ey.crec.cn (Y.G.); dingwy@ey.crec.cn (W.D.)

**Keywords:** cement soil, peat soil environment, corrosion behavior, observation of apparent phenomena, ion leaching, unconfined compressive strength

## Abstract

To study the corrosion behavior of cement soil in peat soil, the experiment involves the preparation of peat soil by incorporating humic acid into cohesive soil with a lower organic matter content. Cement soil samples are then prepared by adding cement to the mixture. These samples are subjected to immersion in fulvic acid solution and deionized water to simulate different working environments of cement soil. The experiment considers immersion time as the variable factor. It conducts observations of apparent phenomena, ion leaching tests, and unconfined compression strength tests on the cement soil. The experiment results are as follows: (1) With increasing immersion time, the surface of the cement soil in the peat soil environment experiences the disappearance of Ca(OH)_2_ and calcium aluminate hydrate. Additionally, large amounts of bird dropping crystals precipitate on the surface and within the specimen. The cement soil undergoes localized disintegration due to extensive erosion caused by swelling forces. (2) In the peat soil environment, fulvic acid reacts with the hydration products of cement, resulting in partial leaching of ions such as Ca^2+^, Mg^2+^, Al^3+^, and Fe^3+^ into the immersion solution. The lower the pH of the fulvic acid immersion (indicating higher concentration), the more significant the ion leaching. Increasing the ratio of humic acid to cement can slow down the leaching of ions. The cement soil undergoes dissolutive erosion in the peat soil environment. (3) The peat soil environment exerts both strengthening and corrosive effects on the cement soil. Cement soil without humic acid exhibits noticeable corrosion in the peat soil environment, gradually decreasing strength as immersion time increases. The strength decreases by 83% from 28 to 365 days. In contrast, cement soil with humic acid experiences an initial period of strengthening, leading to a significant increase in strength in the short term (34% increase from 28 to 90 days). However, the corrosive effects gradually dominate, resulting in a decrease in strength over time. The strength decreases by 80% from 90 to 365 days. This study also explores the strengthening effects of peat soil on cement soil. It identifies phenomena such as extensive erosion and new substance precipitation in cement soil.

## 1. Introduction

Peat soil (also called peat or peaty soil) comprises inorganic minerals, organic matter aggregates, and plant residues [1]. Under suitable climatic and topographic conditions, plant remains undergo oxidation and partial decomposition in wetland swamps to form humus. The humus then binds the undecomposed organic matter residues with the inorganic minerals in the soil, accumulating peat soil over long periods [2]. According to statistics, peat soil is distributed in 59 countries worldwide, covering approximately 5% to 8% of the Earth’s land area. Among them, Canada has the widest distribution of peat soil. China also has abundant peat soil reserves, with a distribution area of approximately 42,000 square kilometers, ranking seventh globally [3]. Various scholars have conducted numerous experimental studies to improve the engineering properties of peat soil. They have incorporated silicate cement into the peat soil layer, thoroughly mixing it to form cement–soil mixing piles. This method effectively increases the bearing capacity of the peat soil layer and reduces the amount of soil settlement [4,5,6]. However, previous research has confirmed [7,8] that humic acid may severely inhibit the hydration process of cement. In addition, humic acid can also encase the particles of the cement hydration products and react with them, delaying the hydration process, eroding the internal structure of the cement soil, increasing its internal porosity, and thereby reducing its mechanical properties. Therefore, exploring the effect of the corrosion behavior of a peat soil environment on cement soil has particular theoretical support and application value for the corrosion research and durability evaluation of cement soil composite foundations in peat soil distribution areas.

According to previous research, the main component of peat soil is organic matter [9,10]. Organic matter contains a large amount of humic acid, the primary substance affecting the engineering properties of cement soil. Many researchers have done much research on this basis. For example, in 1989, Kamon et al. [11] used cement to solidify fluid ooze. They found that its strength decreases with the increase of humic acid. The presence of ettringite improves the strength of the solidified fluid ooze. In 2002, Tremblay et al. [12] studied the effect of organic compounds on the strength of cement soil. They found that humic acid can affect the development of its strength by affecting the pH value of the pore solution. In 2009, Zhu et al. [13] used dredged sludge to investigate the influence of humic acid on hydration products. They found that humic acid reacts with the calcium hydroxide produced by cement hydration, generating soluble compounds that affect the development of solidified material strength. In 2017, Kang et al. [14] added humic acid to dredged sea clay and conducted research using cement as a solidification agent. They found that both cement and humic acid content determine the strength of cement soil. In the above studies, humic acids have adverse effects on cement soil, which hinder the development of cement soil strength or have corrosive effects on cement soil. The above researchers all regarded humic acid as a whole. They ignored that humic acid is a mixture of humic and fulvic acids and humin. The solubility differences of each substance are different. Based on this, some scholars conducted targeted research on the impact of the dissolution characteristics of humic acid and fulvic acid on the engineering properties of soil and cement soil. For example, in 2003, the complexation of uranium by humic acids and fulvic acids was studied to obtain information on uranium binding onto functional groups of humic substances [15]. In 2015, Cao Jing et al. [16] provided particular theoretical support for exploring the effect of cement-strengthening peat soil foundations around Dianchi Lake. They guided the actual project surrounding Dianchi Lake. Wang et al. [17] used fulvic acid to simulate natural organic compounds in dredged sludge 2022 and then selected cement as the solidification agent. The effect of fulvic acid on the strength of cement-solidified dredged sludge was studied through water content tests, hydration heat tests, scanning electron microscopy tests, and thermogravimetric tests on the samples. The results indicate that fulvic acid adsorbs on the surface of cement particles, hindering the contact between cement and pore water and affecting the hydration reaction of cement. In addition, the carboxyl and hydroxyl groups in fulvic acid adsorb calcium ions in pore water through ion coordination, reducing the generation of hydration products and thereby reducing the strength of the solidified product. Lang L [18] simulated the dredged sludge by adding humic acid into the soil and solidifying it with cement, lime, and nano SiO_2_. However, the above studies only considered the effects of humic acid and fulvic acid on cementitious soil separately. They did not consider the combined effect of the two humic acids on cementitious soil.

In summary, numerous scholars and engineers have found that humic acid hurts the development of cement soil strength. However, humic acid has a complex composition, and the different components have varying forms of existence within the soil, potentially leading to significant differences in long-term corrosion effects on cement soil. Furthermore, previous studies have primarily focused on the corrosion effects while giving less consideration to the strengthening effects and potential phenomena such as extensive erosion and the precipitation of new substances in peat soil environments. Therefore, this study addresses these gaps by incorporating humic acid into cohesive soil with a lower organic matter content to create peat soil samples and adding cement to create cement. These samples are then subjected to immersion corrosion in fulvic acid solution and deionized water (immersion method) to simulate different working environments of cement soil. After the immersion corrosion, the samples are analyzed through observations of apparent phenomena, ion leaching tests, and unconfined compression strength tests to investigate the corrosion characteristics of cement soil in peat soil environments. This research provides a foundation for studying corrosion resistance in cement soil under peat soil conditions.

## 2. Materials and Methods

### 2.1. Materials

**Figure 1:**
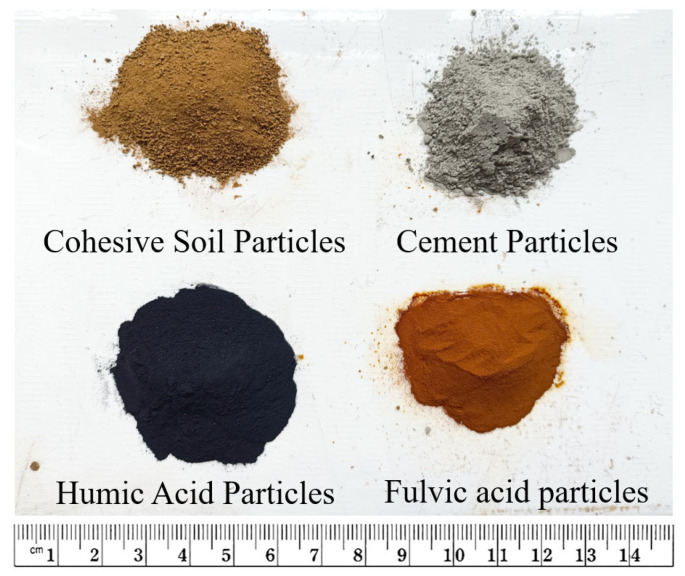
Test materials [19].

The apparent conditions of soil samples, cement, humic acid particles, and fulvic acid powder used in the experiment are shown in Figure 1. Considering the uniformity of material mixing, soil particles will pass through a 2 mm sieve. In contrast, cement particles, humic acid reagent particles, and fulvic acid reagent particles will pass through a 1 mm sieve. Moreover, this method is only used when preparing samples.

The basic information of the test materials is as follows:

(1) Test soil

The soil used for the experiment is alluvial–pluvial cohesive soil, taken from the north slope of Jingyuan Dormitory in Chenggong Campus of Kunming University of Science and Technology, Kunming City, Yunnan Province. After natural air drying, the obtained alluvial–pluvial cohesive soil is finely ground and then sieved using a geotechnical sieve with a diameter of 2.00 mm. It is then sealed and stored in a storage box, and the soil’s particle density (specific gravity) is *ρ*_s *soil*_ = 2.84 g/cm^3^.

The chemical composition and mass fractions of each component of the experimental soil were determined by X-ray fluorescence (XRF). The main chemical components of the soil sample are SiO_2_, Fe_x_O_y_, and Al_2_O_3_. The main phases of the soil sample are quartz, kaolinite, mica, goethite, and anatase, with a single composition and low organic content. Therefore, the impact on the experimental results is relatively small when using this soil as the experimental material to study the corrosion behavior of peat soil environment on cement soil.

(2) Cement

The cement is selected from Shilin brand ordinary Portland cement produced by Yunnan Huaxin Cement Co., Ltd. (Yunnan, China) with a strength grade of 42.5, abbreviated as PO 42.5. The cement’s particle density (specific gravity) is ρs cement = 3.1 g/cm^3^.

(3) Humic acid reagent

Tianjin Guangfu Chemical Reagent Factory made the humic acid reagent used in the experiment. It was determined that the humic acid content in the reagent was 41.68%, and its particle density (specific gravity) ρs acid = 1.85 g/cm^3^.

(4) Fulvic acid reagent

The fulvic acid reagent used in the experiment was a biochemical fulvic acid reagent produced by Pingxiang Red Soil Humic Acid Co., Ltd. (Jiangxi, China) The fulvic acid content in the fulvic acid reagent was determined to be 60%.

(5) Test water

The test water is deionized water.

### 2.2. Experimental Design and Sample Preparation

This experimental plan will focus on the relationship between peat soil’s organic matter content, humic acid content, and the cement rate. The experiment will improve reproducibility through detailed experimental process records, transparent reagent sources and quality assurance, accurate formulas, equipment information, data processing and analysis methods, and clear experimental design tables.

According to research [19], the organic matter content in peat soil in the Dianchi and Erhai regions ranges from 10.73% to 75.09%. The total humic acid content ranges from 7.15% to 50.06%, with humic acid (HA) content ranging from 2.36% to 28.13%. An experimental design was conducted based on the measured values of humic acid in the peat soil of Dianchi Lake mentioned above. To better understand the impact of various humic acid content gradients on cement soil and adapt to engineering practice, the dosage values of humic acid reagents in this experiment are 0%, 15%, 20%, 25%, and 30%. Respectively, the cement mixing ratio is 15%, 20%, and 25%. (The above addition ratios are all calculated based on the weight of the samples.) Moreover, immerse the cement soil sample in a fulvic acid solution (continuously adding fulvic acid to maintain a constant pH value) and deionized water. The soaking time of the cement soil is set to 28 days, 90 days, 180 days, 270 days, and 365 days. To ensure the uniformity and effectiveness of the experimental results, the experiment is conducted with four parallel samples, totaling 800 samples. To meet the sampling conditions and essential characteristics of peat soil, determine and control the moisture content of peat soil samples ω = 24%, void ratio e = 1.2, Sample dimensions: Inner diameter (*d*) = 39.10 mm, height (*h*) = 80.00 mm. The test plan is shown in Table 1. According to the Standard for Soil Test Methods (GB/T50123-2019) [20], the samples were prepared, maintained, and soaked according to existing literature [21].

### 2.3. Testing Process

**Figure 2:**
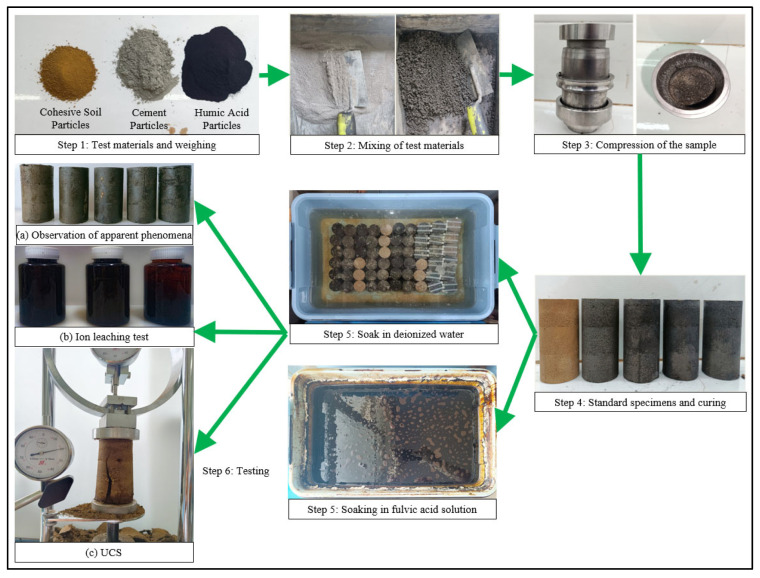
Sample production and testing process.

This article conducts surface observation, an ion leaching test, and an unconfined compressive strength test on the sample. The specific testing process is as follows, and the testing process is shown in Figure 2. First, mix the soil, cement, and humic acid particles evenly and place them in a three-part mold (the same size as the sample). Then, the prepared sample is demolded and soaked in deionized water and fulvic acid solution. Finally, various sub-tests are conducted separately after the sample reaches the soaking age. The above tests are strictly carried out according to the Standard for Soil Test Methods (GB/T50123-2019) [20].

(1) Observation experiment of apparent phenomena

Place the cement soil samples separately in the soaking box, control the liquid level of the saturated solution to be 2.0–2.5 cm above the top of the sample, and continuously add the fulvic acid reagent to the soaking box to maintain the stable pH value of the soaking solution (without adding deionized water for soaking); take photos to record the changes in the apparent phenomena of the samples at each soaking time.

(2) Ion leaching test

Place the cement soil samples in separate soaking boxes, control the liquid level of the saturated solution to be 2.0–2.5 cm above the top of the sample, and continuously add the fulvic acid reagent to the soaking box to maintain the stable pH value of the soaking solution (without adding deionized water for soaking). Take 150 mL of each sample soaking solution with a soaking time of 90 days, and measure the content of Ca^2+^, Mg^2+^, Al^3+^, and Fe^3+^ that significantly impacts the sample’s strength.

(3) Unconfined compressive strength test

The unconfined compressive strength of cement soil samples soaked for 28 days, 90 days, 180 days, 270 days, and 365 days was measured using a lime soil electric unconfined compressive tester (YSH-2 type, Nanjing Ningxi Soil Instrument Co., Ltd., Nanjing, China). The axial compression rate of the experimental control instrument is 1.0 mm/min, and the sample is continuously pressed until it is destroyed. The average strength of the tested sample is taken as the strength of the group of samples.

In addition, this article will also conduct microscopic experimental tests on the corrosion process of cement soil in peat soil environments. The steps are as follows: the cement soil samples soaked in sulfuric acid solution are retrieved, cleaned, and naturally dried. The newly formed products are collected for SEM (VEGA3-TESCAN, Czech Republic) experiments to observe the micro-area morphology of yellow crystals. Point scanning is conducted to perform energy-dispersive spectroscopy (EDS) on the newly formed products in the measurement area. Finally, XRD (Panaco X′pert3 powder X-ray diffractometer, The Netherlands) experiments are conducted to identify their phases.

## 3. Test Results and Analysis

### 3.1. Observation of Apparent Phenomena and Analysis of Ion Leaching Results

A large amount of humic acid makes the peat soil environment acidic, which is detrimental to the development of cement soil strength and has a corrosive effect on cement soil. When cement soil works in a peat soil environment for a long time, the changes in apparent phenomena can directly reflect the severity of its erosion by the peat soil environment. Further ion leaching tests can be conducted to preliminarily determine the type of corrosion of peat soil environment on cement soil.

#### 3.1.1. Observation of Changes in Apparent Phenomena

**Figure 3:**
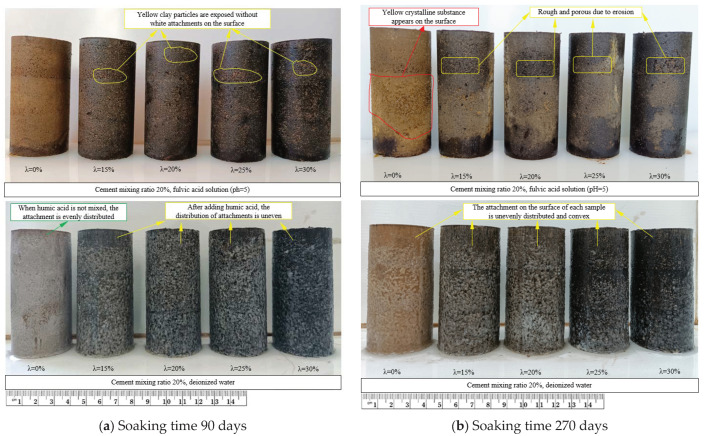

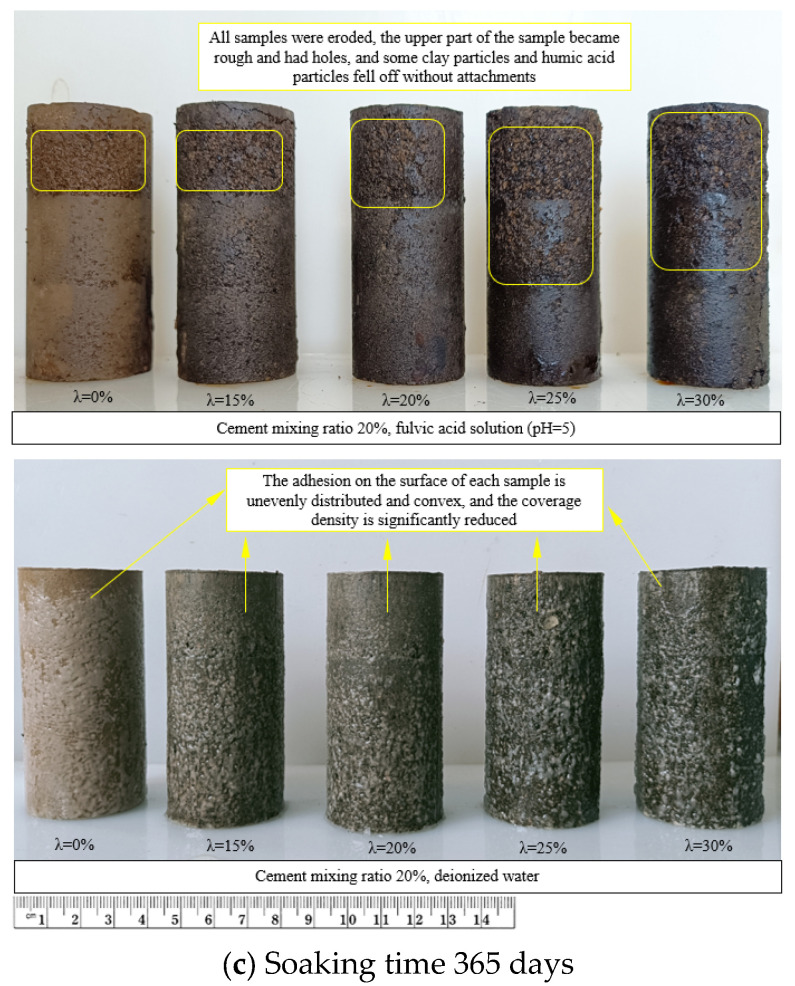
Changes in apparent phenomena of various samples in fulvic acid solution (pH = 5.0) and deionized water with soaking time.

When cement soil is exposed to peat soil for a long time, its erosion varies depending on the environment’s pH value and soaking time. Therefore, a cement mixing ratio of 20% and a pH value of 5 in the fulvic acid soaking solution were selected to observe changes in apparent phenomena. The cement soil samples were observed with soaking time (90 d, 270 d, 365 d) as the change factor, and the cement soil samples soaked in deionized water were used as the control group. The changes in apparent phenomena are shown in Figure 3a–c, and the changes in apparent phenomena are described in Table 2.

**Figure 4:**
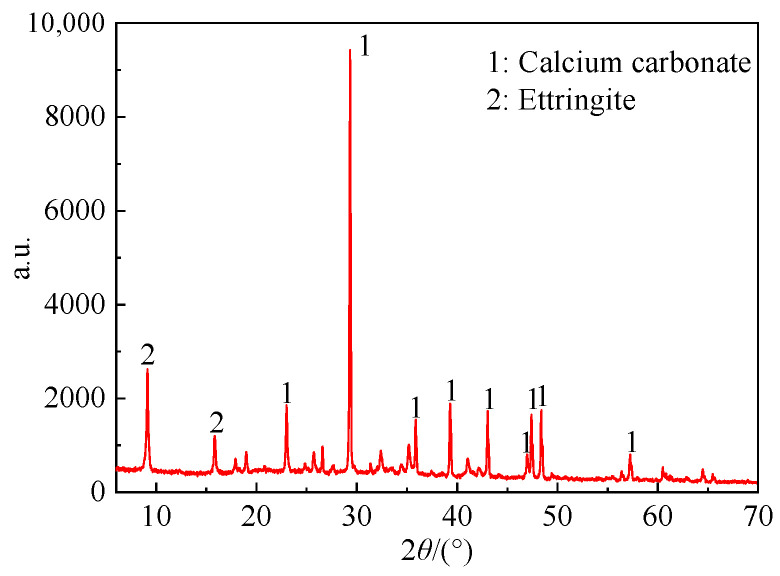
XRD diffraction pattern of white matter on the surface of the sample immersed in deionized water.

The XRD test results of white matter on the sample’s surface soaked in deionized water are shown in Figure 4. Its main components are calcium carbonate (ICDD: 01-086-2334) (CaCO_3_) and ettringite (ICDD: 00-041-1451) (3CaO · Al_2_O_3_ · 3CaSO_4_ · 32H_2_O), which are calcium hydroxide (Ca(OH)_2_) precipitated from cement hydration (reacted with CO_2_ to form CaCO_3_ during natural air drying in the air) and ettringite attached to the surface of the sample. The Ca(OH)_2_ and 3CaO · Al_2_O_3_ · 3CaSO_4_ · 32H_2_O adhered to the surface of the cement soil sample soaked in fulvic acid solution were dissolved, and no white matter was observed. The cement soil sample shows a softening phenomenon due to erosion by the peat soil environment. Yellow clay particles are gradually exposed to the saturated solution, even causing the surface of the cement soil sample to become rough and have holes of varying sizes.

On the other hand, adding humic acid enhances the dispersion of clay particles on the surface, deteriorating cement soil samples’ physical properties and structure [16]. The rate of contact and rotation between fulvic acid solution and cement hydration products increases, and the erosion rate is correspondingly faster. Therefore, when subjected to erosion by the peat soil environment for the same time, the cement soil sample mixed with humic acid first exhibits softening and even disintegration, and the more significant the amount of humic acid added, the more pronounced the softening and disintegration phenomenon. In addition, it can be observed that the upper part of the cement soil sample is severely corroded. In contrast, the lower part is slightly less corroded. Above is because the precipitates in the fulvic acid solution deposit at the bottom and around the cement soil sample, creating a gripping force on the cement soil sample and forming a covering layer on its surface, which has a particular blocking effect on the infiltration of the fulvic acid solution in the cement soil sample and slows down the corrosion rate. Therefore, the lower part of cement soil is slightly less corroded.

**Figure 5:**
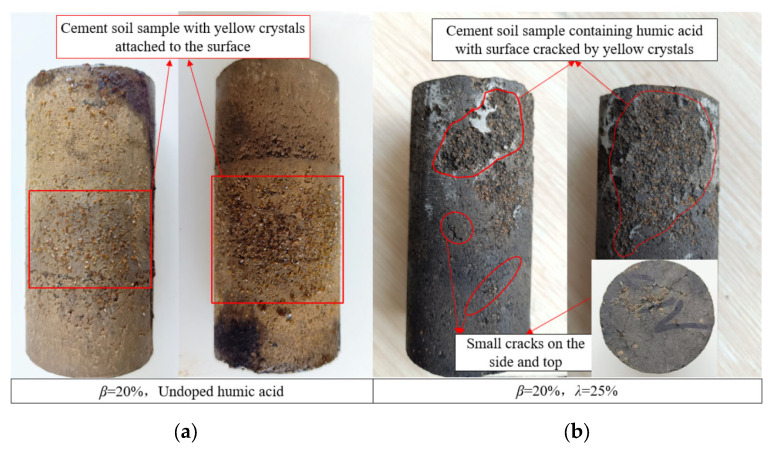
Photos of cement soil sample attached with yellow crystals.

Select a cement soil sample with a pH value of 5, a cement addition ratio of 20%, a soaking time of 270 days, and no humic acid or a 25% addition of humic acid for observation, as shown in Figure 5.

However, with the gradual increase of the cement ratio, cement hydration products increase. In addition to cement soil particles, hydration products gradually fill the pores of cement soil samples so that the influence of HA on cement soil samples is gradually reduced. The cement soil sample without humic acid has dense yellow crystals attached to its surface, and the sample is relatively intact (Figure 5a). The surface area of the cement soil sample mixed with humic acid becomes very rough, with small and unconnected cracks visible (Figure 5b). After the location of the minor cracks is lifted, the developed yellow crystals can be seen. The surface of the cement soil sample is filled with the developed yellow crystals and undergoes expansion cracking. After the cracks (expansion-type erosion) are generated, the surface layer falls off and forms a rough surface.

**Figure 6:**
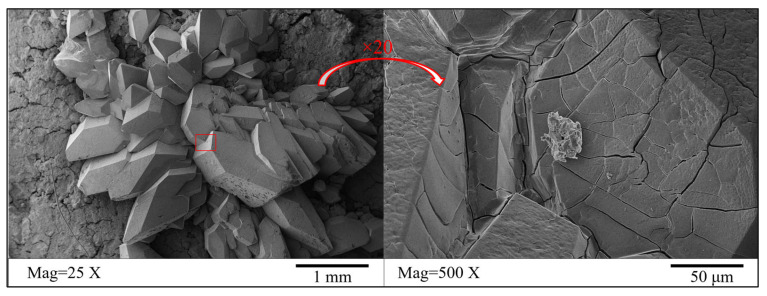
SEM image of yellow crystals on the surface of the sample soaked in fulvic acid solution.

**Figure 7:**
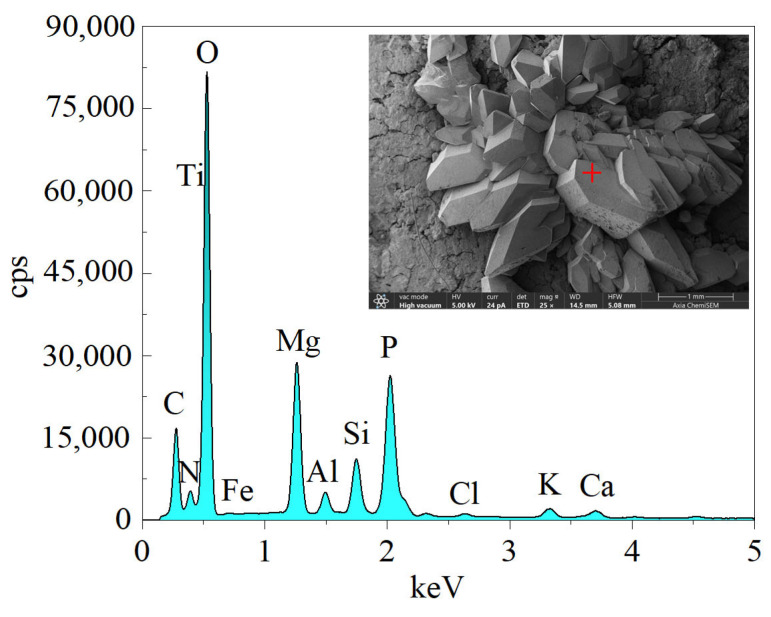
EDS diagram of yellow crystals on the surface of the sample soaked in fulvic acid solution.

**Figure 8:**
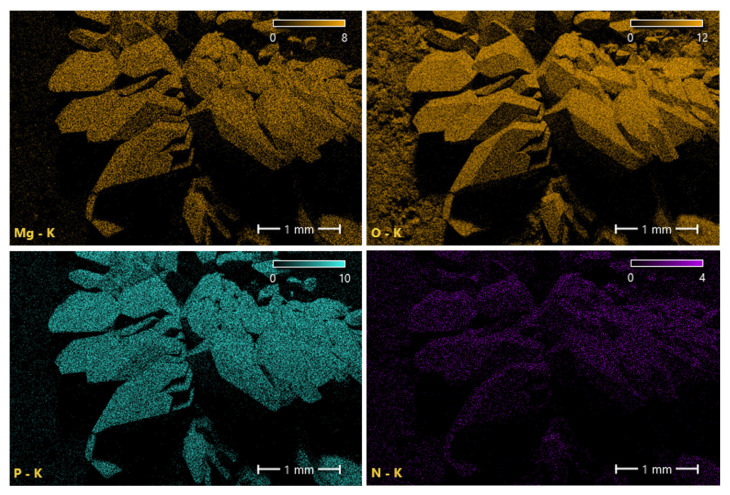
Distribution of main elements in the yellow crystal measurement area of the sample surface soaked in fulvic acid solution.

Take the yellow crystals on the sample’s surface without humic acid doping for SEM testing to observe the microstructure of the yellow crystals, as shown in Figure 6. Before conducting SEM testing on the sample, in order to increase its conductivity, it is necessary to spray gold (Au) treatment on it. Moreover, energy dispersive spectroscopy (EDS) was performed on the yellow crystals in the measurement area using a point scanning method, as shown in Figure 7. This article will use EDS analysis in Auto Mode. In automatic mode, the system will automatically scan the entire sample or selected area and generate an X-ray spectrum. The distribution of the main elements within the survey area is shown in Figure 8. SEM experiments showed that the yellow crystal exhibited axial, wedge-shaped, short columnar, and thick plate-like aggregates. When the local position was magnified 500 times, there were a few crisscrossing cracks in the crystal under the electron microscope. EDS experiments showed that the main elements in the yellow crystal measurement area are C, N, O, Mg, P, and Si. The content of the O element is the most abundant among non-metallic elements, while the Mg element is the most abundant among metallic elements. The element distribution map shows that both Mg and P elements are only distributed in the area where the yellow crystal is located. In contrast, O and N elements are evenly distributed throughout the entire measurement area, indicating that Mg and P elements are unique components of the yellow crystal. EDS analysis showed the presence of metallic and non-metallic elements, indicating that a series of complex corrosive chemical reactions occurred when the sample was prepared and immersed in a fulvic acid solution. The humic acid dissolved in water interacted with various ions in the solution, causing precipitation and recrystallization on the surface and inside the cementitious soil sample to form a rhombic crystal.

**Figure 9:**
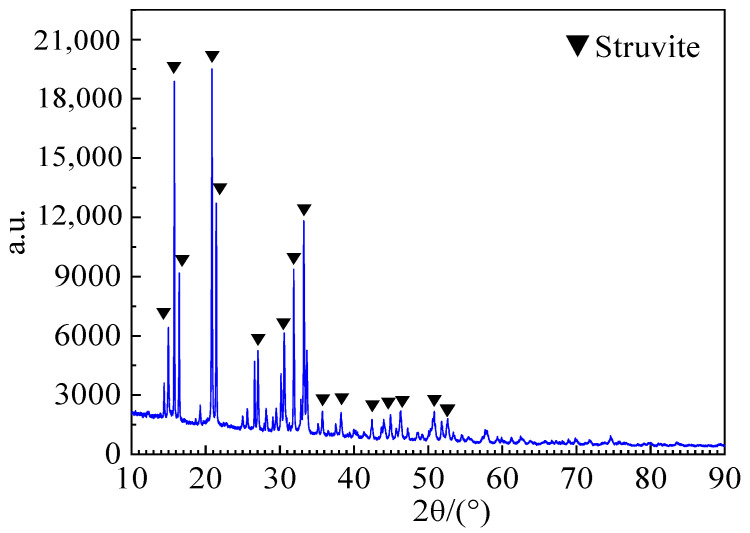
XRD diffraction pattern of yellow crystals on the surface of the sample soaked in fulvic acid solution.

To further clarify the main components of yellow crystals, the cement soil sample soaked in fulvic acid solution was removed, cleaned, and naturally dried. The yellow crystals on the sample’s surface were collected for XRD testing to identify their phase, and the results are shown in Figure 9. The main component of this yellow crystal is struvite (ICDD: 98-000-0419), with a chemical formula of Mg (NH_4_) PO_4_ · 6H_2_O. Struvite is mainly a rhombic crystal formed by the interaction between humic acid dissolved in water and various ions in the solution, which precipitates and recrystallizes on the surface and inside of the cement soil sample. The formation of this substance fills the pores of the cement soil sample. However, as the soaking time increases, the crystal volume inside the sample gradually increases. The cement soil sample undergoes extensive erosion due to the expansion effect of the crystals.

By analyzing and comparing the changes in the apparent phenomena of the cement soil samples in the figure, it can be found that the cement soil samples without humic acid only form struvite crystals on the surface, and the samples are relatively intact. After adding humic acid, the cement soil sample formed struvite crystals inside and outside. Above is because the growth and development of the crystals require a large amount of growth space. After adding humic acid, the dispersion of the clay particles inside the cement soil is enhanced. The pore connectivity formed is robust, providing space for the growth and development of struvite crystals, causing the surface of the cement soil sample to experience expansion and cracking.

#### 3.1.2. Analysis of Ion Leaching Test Results

According to previous studies, when humic acid reacts with alkali metal hydroxide (such as Ca (OH)_2_, Mg(OH)_2_, Al(OH)_3_), the bivalent salt generated is insoluble. The trivalent salt generated is soluble, and when fulvic acid reacts with alkali metal hydroxide, the salt generated is soluble [22].

When the cement soil sample is immersed in a fulvic acid solution, the peat soil environment comes into contact with the cement soil. It reacts, resulting in ion exchange between the cement and peat soil environments, leading to the leaching of various metal cations.

When studying the effect of humic acid addition on ion leaching, the fixed cement addition ratio was 20%, the pH value of the fulvic acid solution was 5, and the soaking time was 90 days. The results are shown in Table 3. As the amount of humic acid added increases, the mass concentration of each ion in the saturated solution of the cement soil sample shows a roughly decreasing trend. The above is because humic acid particles have a loose and porous sponge-like structure, with strong water retention and holding capacity and strong adsorption and accommodation capacity for small fulvic acid molecules. When a large amount of humic acid is added to the cement soil, the fulvic acid immersed in the sample is heavily adsorbed. It does not react with the cement hydration products promptly, affecting the leaching of various metal cations.

When studying the pH value of the soaking solution as a factor affecting ion leaching, the fixed cement addition ratio was 20%, the humic acid addition amount was 20%, and the soaking time was 90 days. The results are shown in Table 4. As the pH value of the soaking solution decreases, the mass concentrations of Mg^2+^, Al^3+^, and Fe^3+^ in the soaking solution gradually decrease. The mass concentration of Ca^2+^ in the soaking solution with a pH value of 6 is slightly higher than that in the soaking solution with a pH value of 5. However, the amount of Ca^2+^ dissolved in the peat soil environment is 4.1–5.6 times that of deionized water. Overall, the erosion of the peat soil environment causes a large amount of Ca^2+^ to dissolve. The mass concentration of each metal cation in the fulvic acid soaking solution is much higher than that in the deionized water soaking solution because the fulvic acid is easy to dissolve in water and ionize a large number of hydrogen ions, which react with alkali metal hydroxides such as Ca(OH)_2_ produced by cement hydration. Some hydration products on the surface and inside the cement soil sample dissolve, and each metal cation rapidly dissolves into the saturated solution. The above increases the mass concentration of metal cations in the soaking solution.

When studying the effect of cement addition ratio on the leaching of various metal cations, the fixed amount of humic acid added was 20%, the pH value of the soaking solution was 5, and the soaking time was 90 days. The results are shown in Table 5. The mass concentration of each ion in the saturated solution of the sample without cement is the lowest, and the mass concentration of each metal cation after adding cement is higher than that of the sample without cement. The above is because after adding cement, the cement undergoes a hydration reaction, and its hydration products react with fulvic acid and dissolve into the fulvic acid soaking solution. After adding cement, the mass concentration of each ion in the soaking solution (pH = 5) of the cement soil sample decreases with the increase of the cement addition ratio. The mass concentration of each ion in the saturated solution of the cement soil sample with a cement addition ratio of 25% is the lowest. The above is because a higher cement addition ratio results in a better filling effect of the formed hydration products on the pores of the cement soil sample. The migration of fulvic acid solution in the cement soil sample is less accessible, and the rotation speed is slower, which can only slowly erode inward. The mass concentration of various metal cations in the soaking solution is low.

### 3.2. The Change Behavior and Analysis of Cement Soil Strength under the Corrosion of Peat Soil Environment

**Figure 10:**
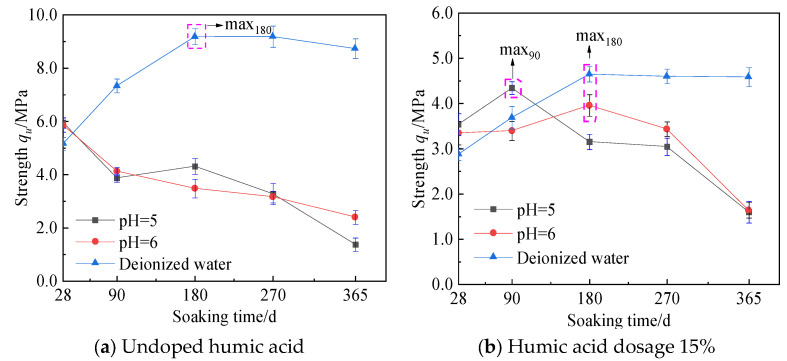

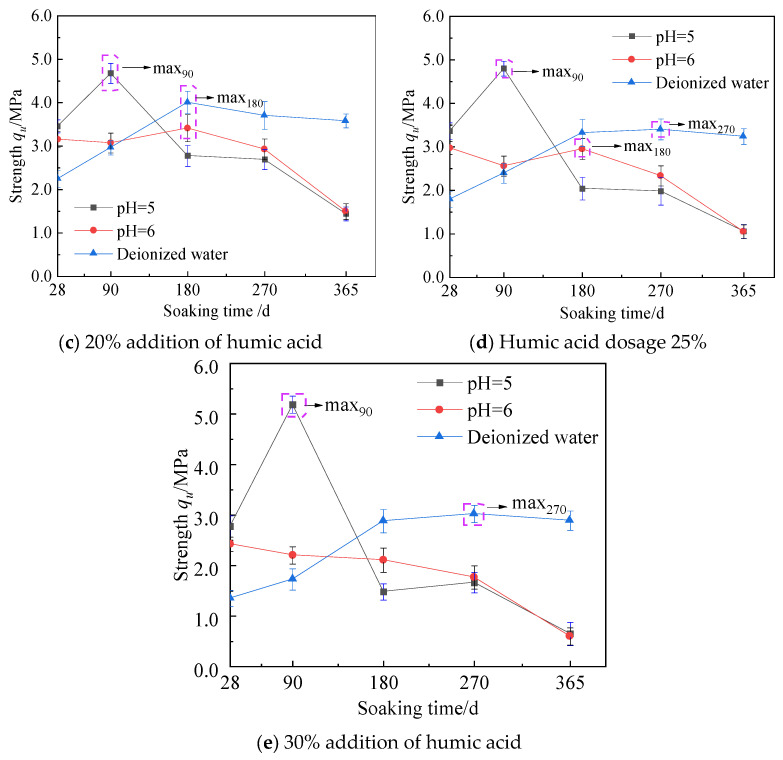
Relationship curve between strength and soaking time of cement–soil samples with a cement content of 20%.

To investigate the corrosion behavior of cement soil in a peat soil environment during long-term service, a 20% cement soil sample was selected for the study. Figure 10a–e shows the relationship curve between strength and soaking time.

When the cement soil sample is immersed in deionized water, its strength gradually increases with the soaking time and then slightly decreases. As the soaking time increases, the cement hydration reaction gradually proceeds, and the hydration products gradually increase, leading to a significant increase in the strength of cement soil. However, under the long-term infiltration of free water, the cement hydration reaction of cement soil tends to complete, and humic acid consumes some of the cement hydration products [16]. Moreover, water infiltration gradually softens the cement soil, resulting in a slight decrease in strength.

When the cement soil samples are immersed in fulvic acid solution, the strength of the samples without humic acid decreases with increasing immersion time. The strength decreases by 83% from 28 to 365 days. On the other hand, the strength of the cement soil samples with humic acid initially increases gradually with immersion time, with a 34% increase in strength from 28 to 90 days. However, the strength gradually decreases, with an 80% decrease from 90 to 365 days. Furthermore, the samples immersed in fulvic acid solution with a pH value of 5 experienced a more significant decrease in strength. At a particular time within the soaking time range of 90 to 180 days, the strength of cement soil containing humic acid is lower than that soaked in deionized water, and the lower the pH value (higher concentration) of the fulvic acid soaking solution, the lower the strength. The peat soil environment has both strengthening and corrosion effects on cement soil, which run through the entire soaking process. However, the dominant roles of strengthening and corrosion differ at different soaking times. In the early stage of soaking (before 90 days), the strength enhancement effect of the fulvic acid solution on cement soil containing humic acid is dominant. Specifically, the lower the fulvic acid soaking solution’s pH value (the higher the concentration), the more pronounced the strengthening effect and the higher the strength. However, in the long run, the corrosion effect of the fulvic acid solution on cement soil is dominant, specifically manifested as the lower the pH value (higher concentration) of the fulvic acid soaking solution, the longer the soaking time, the more pronounced the corrosion effect on cement soil, and the lower the strength.

The strength change of cement soil exhibits the above pattern because humic acid has a sponge-like structure and strong water retention capacity. The maximum water absorption can exceed 500% [22], accommodating a large amount of fulvic acid. Fulvic acid has colloidal properties [23,24,25], bonding on cohesive soil particles and a particular filling effect on the pores of cement soil. Therefore, even with ion leaching, the strength of cement soil with the exact dosage of humic acid still increases. However, the molecular structure of fulvic acid contains a large number of acidic functional groups, such as the carboxyl group and phenolic hydroxyl group, which easily ionize a large number of hydrogen ions after dissolving in water, making the saturated solution acidic and eroding the cement soil. As the soaking time increases, the cement hydration reaction gradually proceeds, and the cement hydration tends to be completed. The corrosion effect of fulvic acid on the cement soil is dominant, and the strength of the cement soil decreases sharply. The peat soil environment shows a significant corrosion effect on the cement soil.

During the soaking process, firstly, the strengthening effect of the peat soil environment on cement soil dominates. Then, the corrosion effect on cement soil dominates. In the long run, when a peat soil environment erodes cement soil, its strength decreases, which may lead to failure to meet practical engineering needs and even collapse and complete loss of bearing capacity. In addition, when the strength of cement soil begins to decrease, the corrosive effect of the peat soil environment on cement soil is very prominent, and the strength decreases very quickly. However, cement soil’s strength reduction rate slows as the soaking time increases. The reason is that when peat soil infiltrates cement soil, the infiltration rate of acidic corrosive substances in cement soil gradually slows as the infiltration depth increases. Secondly, the hydration products are entirely consumed, and the rate of strength reduction slows down.

## 4. Conclusions

To explore the corrosion behavior of peat soil environment on cement soil, this article made a preliminary judgment on the corrosion type of cement soil through observation tests of apparent phenomenon changes and ion leaching tests, and then directly characterized the corrosion behavior of peat soil environment on cement soil through the unconfined compressive strength of cement soil. The conclusions drawn from the study are as follows:

(1) With the increase of soaking time, a large amount of Ca(OH)_2_ and ettringite appear on the surface of cement soil soaked in deionized water. No white calcium hydroxide and ettringite are observed on the surface when the cement soil is used in the peat soil environment. However, struvite crystals are observed to precipitate, and the cement soil has extensive erosion.

(2) Fulvic acid reacts with cement hydration products, which makes Ca^2+^, Mg^2+^, Al^3+^, and Fe^3+^ plasma dissolve in the immersion solution. The lower the pH value of the fulvic acid immersion (the higher the concentration), the more pronounced the dissolution of metal cations. The increase of humic acid can slow down the dissolution of ions. Increasing the cement mixing ratio will also slow down the infiltration rate of fulvic acid solution in cement soil and then slow down the leaching of metal cations. The dissolution of metal cations indicates that the corrosion of cement soil occurs.

(3) The peat soil environment simultaneously exerts both strengthening and corrosive effects on the cement soil throughout the immersion process. Cement soil without humic acid experiences more noticeable corrosion in the peat soil environment, gradually decreasing strength as immersion time increases. The strength decreases by 83% from 28 to 365 days. On the other hand, cement soil with humic acid initially experiences a dominant strengthening effect in the peat soil environment, leading to a substantial increase in strength in the short term. From 28 to 90 days, the strength increases by 34%. However, the corrosive effects gradually take over, resulting in a gradual decrease in strength over time. From 90 to 365 days, the strength decreases by 80%.

(4) After being eroded by the peat soil environment for a long time, the strength of cement soil gradually decreases, even collapses, and ultimately loses its bearing capacity, which cannot meet the actual needs of the project. Therefore, cheap and efficient admixtures can be selected to enhance its structure, slow down the infiltration rate of fulvic acid and other corrosive substances in cement soil, and prevent the formation of struvite to prevent extensive erosion of cement soil.

(5) In comparison to previous research findings [26,27,28], this study observed the occurrence of extensive erosion in cement soil in the peat soil environment, and the deposition of bird guano crystals was also discovered. Additionally, existing research primarily focused on the corrosion effect on cement soil. At the same time, this study emphasized the dual impact of the peat soil environment, involving both corrosion and strengthening phenomena. These findings are of significant importance for a deeper understanding of the corrosion patterns of cement soil in peat soil environments and provide new guidance for implementing appropriate measures to prevent corrosion damage.

## Figures and Tables

**Table 1:** Test plan.

Test Items	Cement Ratio β/%	Humic Acid Content λ/%	Soaking Time/d	Type of Soaking Solution
Observation of apparent phenomena	20	0, 15, 20, 25, 30	90, 270, 365	FA solution (pH = 5), Deionized water
Ion leaching test	20	0, 15, 20, 25, 30	90	FA solution (pH = 5)
	20	20	90	FA solution (pH = 5), Deionized water
	0, 15, 20, 25	20	90	FA solution (pH = 5)
UCS	20	0, 15, 20, 25, 30	28, 90, 180, 270, 365	FA solution (pH = 5), Deionized water

**Table 2:** Changes in the apparent phenomena of the samples.

Soaking Time/d	Soak in Deionized Water with a Cement Content of 20%	Soak in Fulvic Acid Solution (pH = 5.0) with a Cement Content of 20%
90	There are more white attachments on the surface of the sample. The white attachments on the sample’s surface without humic acid are evenly distributed. However, the distribution of white substances on the sample’s surface with humic acid is uneven. It decreases with the increase in the amount of humic acid.	Each cement soil sample is relatively complete. There is no white attachment on the surface of each sample. The surface of the sample without humic acid has no noticeable change. In contrast, the sample’s surface with humic acid has become relatively rough, the hole at the contact surface has expanded due to the erosion of the peat soil environment, and the surface of the eroded sample shows light yellow clay particles.
270	The white attachments on the surface of samples with different humic acid contents were unevenly distributed and convex. The white attachment on the surface was significantly reduced compared to 90 days and gradually decreased with the increased amount of humic acid.	Each cement soil sample was damaged to varying degrees. There is no white attachment on the surface of each sample. The surface of the sample without humic acid was attached with a light-yellow crystalline substance, and the bottom was missing an angle. The hole at the contact surface of the sample is further expanded, and the phenomenon is more evident with the increase of the amount of humic acid.
365	White attachments are unevenly distributed on the sample’s surface and are convex. Compared with the other soaking time, the white attachment on the sample’s surface doped with humic acid decreased significantly, the coverage density decreased, and the black humic acid particles were exposed. However, the white attachment on the surface of cement soil without humic acid has no apparent change.	The cement soil samples with different humic acid content were eroded to varying degrees. No white attachment was observed on the surface of the sample. The corrosion degree of the samples without humic acid is weak. However, with the increase in the amount of humic acid, the corrosion degree of the samples is more serious, and the surface becomes rough due to spalling. It can be observed that the erosion of the peat soil environment on the sample is from top to bottom.

**Table 3:** Mass concentrations of various ions under different amounts of humic acid addition (β = 20%, pH = 5, 90 d).

Humic Acid Content/%	Ion Mass Concentration/(mg·mL^−1^)			
	Ca^2+^ (±0.02‰)	Mg^2+^ (±0.02‰)	Al^3+^ (±0.009‰)	Fe^3+^ (±0.1‰)
0	206.60	139.10	2233.65	191.60
15	178.00	124.50	2138.90	168.00
20	181.80	122.70	2084.38	165.50
25	166.20	113.20	1854.67	152.20
30	189.60	117.40	1807.03	156.60

**Table 4:** Mass concentration of various ions under the influence of the pH value of the soaking solution (β = 20%, λ = 20%, 90 d).

Type of Soaking Solution	Ion Mass Concentration/(mg·mL^−1^)			
	Ca^2+^ (±0.02‰)	Mg^2+^ (±0.02‰)	Al^3+^ (±0.009‰)	Fe^3+^ (±0.1‰)
Fulvic acid solution (pH = 5)	181.80	122.70	2084.38	165.50
Fulvic acid solution (pH = 6)	232.10	75.30	295.61	20.16
Deionized water	41.34	1.30	3.36	0.04

**Table 5:** Mass concentrations of various ions under different cement admixture ratios (pH = 5, λ = 20%, 90 d).

Cement Mixing Ratio/%	Ion Mass Concentration/(mg·mL^−1^)			
	Ca^2+^ (±0.02‰)	Mg^2+^ (±0.02‰)	Al^3+^ (±0.009‰)	Fe^3+^ (±0.1‰)
0	101.80	88.69	1050.66	100.20
15	204.00	128.00	2078.03	174.00
20	181.80	122.70	2084.38	165.50
25	167.40	117.00	1876.90	157.10

## Data Availability

The data used to support the finding of this study are included in the article.
